# Influenza Virus Genomic Surveillance, Arizona, USA, 2023–2024

**DOI:** 10.3390/v16050692

**Published:** 2024-04-27

**Authors:** Rabia Maqsood, Matthew F. Smith, LaRinda A. Holland, Regan A. Sullins, Steven C. Holland, Michelle Tan, Gabrielle M. Hernandez Barrera, Alexis W. Thomas, Mario Islas, Joanna L. Kramer, Lora Nordstrom, Mary Mulrow, Michael White, Vel Murugan, Efrem S. Lim

**Affiliations:** 1 Center for Fundamental and Applied Microbiomics, Biodesign Institute, Arizona State University, Tempe, AZ 85281, USA; 2 Virginia G. Piper Center for Personalized Diagnostics, Biodesign Institute, Arizona State University, Tempe, AZ 85281, USA; 3 Arizona State University Health Services, Tempe, AZ 85281, USA; 4 Division of Primary, Complex, and Adolescent Medicine, Phoenix Children’s Hospital, Phoenix, AZ 85016, USA; 5 Valleywise Health Medical Center, Phoenix, AZ 85008, USA; 6 School of Life Sciences, Arizona State University, Tempe, AZ 85281, USA; 7 National Centre for Infectious Diseases, Singapore 308442, Singapore

**Keywords:** influenza virus, genomic surveillance, Arizona

## Abstract

Influenza viruses are constantly evolving and are therefore monitored worldwide in the hope to reduce the burden of disease by annual updates to vaccine recommendations. We conducted genomic sequencing of 110 influenza A and 30 influenza B viruses from specimens collected between October 2023 and February 2024 in Arizona, USA. We identified mutations in the hemagglutinin (HA) antigenic sites as well as the neuraminidase (NA) gene in our samples. We also found no unique HA and NA mutations in vaccinated yet influenza-infected individuals. Real-time genomic sequencing surveillance is important to ensure influenza vaccine effectiveness.

## 1. Introduction

Influenza viruses are common causes of acute respiratory infections that result in significant morbidity and mortality annually [1]. The influenza virus genome comprises eight negative-sense, single-stranded RNA segments. The antigenicity of HA and NA surface glycoproteins are important for immunity and are the basis for subtype classification. HA and NA gene mutations are of interest as they can result in phenotypic effects such as viruses escaping host antibodies, acquiring drug resistance, and increasing virulence [2]. The continued selection of amino acid mutations drives the evolution of influenza virus to undergo antigenic drift [3]. As a result, virus lineages selected for influenza vaccines must be regularly updated for optimal vaccine effectiveness [4]. Annual surveillance and estimates of disease burden also reinforce the ever-changing facet of influenza viruses [5].

Genomic surveillance of contemporary influenza viruses in real-time plays an important role for public health. Global Influenza Surveillance and Response System (GISRS) by the World Health Organization (WHO) monitors for circulating influenza virus strains with the collaboration of 112 countries [6]. Local surveillance provides key insights into disease transmission within communities. Influenza viruses subtyping is conventionally performed using real-time RT-PCR (RT-qPCR) assays. While cost-effective and fast, interpretation of PCR assay results is limited to detecting presence/absence and their quantification. Advances in whole genome sequencing and bioinformatics methodologies allow for more detailed genomic characterization beyond simple subtype classification [7,8]. Pathogen genome sequences can reveal novel insights into the evolution, diversity, mutations, and phylogenetic relationships of influenza viruses. Hence, pathogen genome sequencing is becoming integral to public health surveillance, preparedness and response. During the COVID-19 pandemic, pathogen genome sequencing was successfully deployed both on a local and national scale to track the evolution of SARS-CoV-2 variants [9]. Such local sequencing infrastructure can be leveraged towards other public health threats beyond SARS-CoV-2.

In the prior 2022–2023 season, influenza A(H3N2) virus was the dominant virus subtype that circulated in the United States, over influenza A(H1N1)pdm09 and influenza B/Victoria [10]. In this study, we describe the genomic sequencing surveillance of influenza viruses during the 2023–2024 season from local ambulatory settings in Arizona, USA. Using whole genome sequencing, we identified multiple non-synonymous mutations in the HA and NA genes of circulating influenza viruses in comparison to the WHO vaccine strains.

## 2. Materials and Methods

### 2.1. Study Population

Symptomatic individuals seeking care for an acute respiratory infection of ≤7 days duration at ambulatory settings in Arizona (Arizona State University’s Health Services, Phoenix Children’s Hospital and Valleywise Health) were recruited in this study. Patients with illness duration >7 days at the time of respiratory specimen collection were excluded from this study. Study participants (or the individual’s parent/guardian) provided informed written or verbal consent for study enrollment.

### 2.2. Sample Processing and Testing

Respiratory specimens (nasopharyngeal swabs and throat swabs combined) were collected from individuals. RNA was extracted using the KingFisher Flex System (Thermo Fisher Scientific, Waltham MA, USA) according to manufacturer guidelines. RNA extracts were tested for SARS-CoV-2, influenza A and influenza B viruses using RT-qPCR multiplex assays (Applied Biosystems TaqPath COVID-19, FluA, FluB Combo Kit) using the following reaction protocol for a 384-well plate. The 20 µL reaction mixture contained 5 µL of Taqpath 1-step Multiplex Master Mix, 1 µL of TaqPath COVID-19, FluA, FluB RT-PCR Assay Multiplex, and 14 µL of RNA, with a 10% overage calculated in. MS2 phage was assessed as an internal control along with an extraction negative control. The cycling conditions were 25° for 2 min, 53° for 10 min, 85° for 10 min, 95° for 2 min, and 46 cycles of 95° for 3 s and 60° for 30 s.

### 2.3. Next Generation Sequencing

Influenza-positive specimens with RT-qPCR assay cycle threshold values of less than 30 were selected for whole genome sequencing. Libraries were prepared using either an amplification- or hybridization-based approach. In the amplification-based approach, viral RNA underwent mRT-PCR reactions targeting the universal termini of Influenza A or Influenza B genome segments. mRT-PCR was performed as described [11] with minor adaptations utilizing three general-purpose RT-PCR reagents from the Illumina COVIDSeq kit. Briefly, Influenza A mRT-PCR reactions were performed in a total volume of 25 μL consisting of 21.5 μL Influenza A mRT-PCR master mix and 3.5 μL extracted RNA. Influenza A mRT-PCR master mix was generated using 9 μL Illumina FSM (First Strand Mix), 1 μL Illumina RVT (Reverse Transcriptase), 15.6 μL IPM (Illumina PCR Mix), and 1.25 μL 10 μM Influenza A “Uni” termini primer pool. Influenza B mRT-PCR reactions were also performed in a total volume of 25 μL consisting of 21.5 μL Influenza B mRT-PCR master mix and 3.5 μL extracted RNA. Influenza B mRT-PCR master mix was generated using 9 μL Illumina FSM (First Strand Mix), 1 μL Illumina RVT (Reverse Transcriptase), 15.6 μL IPM (Illumina PCR Mix), and 2 μL 10 μM Influenza B “Uni” termini primer pool. Thermal cycling conditions were carried out as previously described. Following mRT-PCR reactions, libraries were prepared from amplicons by on-bead tagmentation using the Illumina COVIDSeq library preparation workflow. In the hybridization-based approach, libraries were constructed from extracted RNA using Illumina RNA Prep with Enrichment with the Respiratory Virus Oligo Panel v2. Libraries were sequenced on the Illumina NextSeq2000 instrument (Illumina, San Diego CA, USA) using 2 × 150 paired-end reads.

### 2.4. Sequencing Analysis

Sequencing reads were trimmed with Trim Galore version 0.6.10 [12] and mapped against a custom database of influenza reference genome sequences using bwa with default parameters [13] (EPI_ISL_105896, EPI_ISL_12799972, EPI_ISL_158598, EPI_ISL_16215828, EPI_ISL_17054792, EPI_ISL_17368133, EPI_ISL_17468448, EPI_ISL_17539974, EPI_ISL_17625905, EPI_ISL_189340, EPI_ISL_189509, EPI_ISL_189522, EPI_ISL_189538, EPI_ISL_189701, EPI_ISL_189706, EPI_ISL_189714, EPI_ISL_200927, EPI_ISL_201365, EPI_ISL_205132, EPI_ISL_253575, EPI_ISL_327591, EPI_ISL_466695, EPI_ISL_96113). The custom database references included influenza strains with different combinations of H(1–18) and N(1–11) genes, influenza B (Victoria and Yamagata), and influenza C, and were downloaded from GISAID (downloaded November 2023). Per sample, mapped reads to database were used to assemble consensus sequences for each gene segment with a minimum depth threshold of 10 and quality filter of 20. Sequences were compared to the cell culture and recombinant-based WHO vaccine reference sequences influenza A/Wisconsin/67/2022(H1N1)pdm09 (OQ203982), influenza A/Darwin/6/2021(H3N2) (OQ718999), and influenza B/Austria/1359417/2021 (B/Victoria) (EPI_ISL_2378894). To facilitate sequence comparisons with other resources, amino acid residues were numbered according to the recommended numbering scheme for influenza HA subtypes [14].

### 2.5. Phylogenetic Analysis

From the assembled consensus genes, we selected all A(H1N1)pdm09, A(H3N2), and A/Victoria sequences with at least 89% nucleotides across the HA gene. We downloaded 2023–2024 season circulating influenza strain datasets (MW626062, EPI1857216, KX058844) from nextclade/nextstrain [15]. These datasets used recent reference sequences and were suitable for the analysis of current viruses. Using MAFFT [16], we then aligned all sequences, including WHO vaccine references (OQ203982, OQ718999, EPI_ISL_2378894) and trimmed the ends for a clean alignment. Finally, we used IQ-TREE [17] with its default settings to derive the optimal trees for A(H1N1)pdm09, A(H3N2), and B/Victoria influenza viruses.

### 2.6. Protein Structure Modeling

HA gene sequences were analyzed using the FluSurver (v1) mutations tool [18] that predicts mutation effects. HA mutations predicted to be of high phenotypic consequence (levels 2 and 3) were mapped to the influenza HA protein crystal structures (PDB: 7KNA, 4WE8, 4FQM). High phenotypic mutations have previously been reported to affect virulence, antigenic drift, drug resistance, host specificity shift, and escape mutants [18]. The antigenic sites on HA, as defined in [19], were overlaid onto protein structures. Structure models were visualized and manipulated using PyMOL.

## 3. Results

### 3.1. Genomic Surveillance 2023–2024

**Figure 1:**
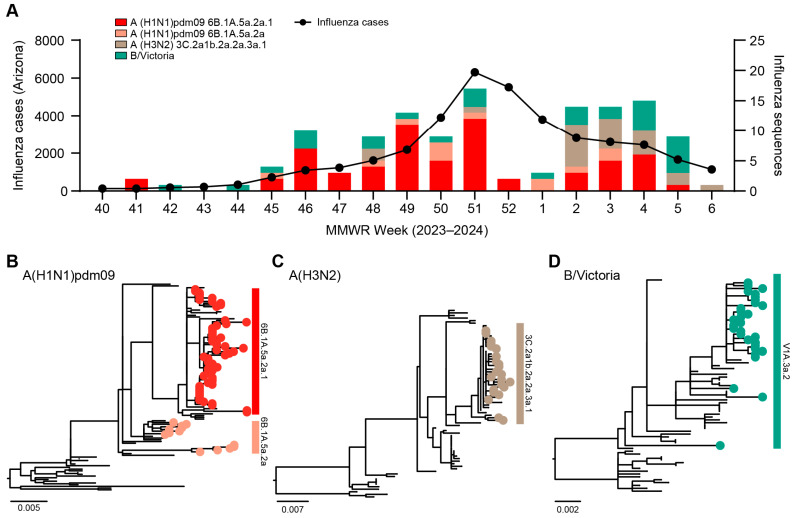
Genomic sequencing analysis of influenza viruses in Arizona, USA, 2023–2024. (**A**) Five-week moving average of laboratory-confirmed influenza cases in Arizona reported by the Arizona Department of Health Services, and influenza genome sequence counts by subtype/clade obtained for specimens used in this study. CDC-established MMWR weeks are standardized numeric labels assigned to weeks that allow for aggregated reporting of disease incidence at the national level. MMWR weeks always begin on a Sunday, and the first MMWR week is the first week of the year which has at least 4 days in a calendar year. Phylogenies of (**B**) influenza A(H1N1)pdm09, (**C**) A(H3N2), and (**D**) B/Victoria viruses HA gene are shown. Arizona sequences from this study are indicated.

To monitor influenza viruses circulating in Arizona, USA, we conducted genomic sequencing of influenza viruses in the 2023–2024 season (Figure 1A). As part of ongoing vaccine effectiveness studies, symptomatic individuals seeking care for an acute respiratory infection of ≤7 days duration at ambulatory settings in Arizona (Arizona State University’s Health Services, Phoenix Children’s Hospital, and Valleywise Health) were recruited. Respiratory specimens (nasopharyngeal swabs and throat swabs combined) were tested for SARS-CoV-2, influenza A, and influenza B viruses using real-time RT-PCR (RT-qPCR) multiplex assays. Influenza-positive specimens with RT-qPCR assay cycle threshold values of less than 30 were selected for whole genome sequencing. The average sequencing depth was 10.6 million paired-end reads per specimen. We assembled the complete/near-complete genome sequences of 110 influenza A virus and 30 influenza B virus cases.

The median age among sequenced cases was 21 (range 0.9–74) years; 66 (47.1%) were male and 74 (52.9%) were female; 58 (41.4%) identified as Hispanic or Latino, 76 (54.3%) as not Hispanic or Latino, and 6 (4.3%) responded with “Don’t know” or “Prefer not to answer”. Specimen collection dates ranged from 12 October 2023 to 5 February 2024. The most frequent symptoms reported were fatigue (89.3%, *n* = 125), runny nose or nasal congestion (88.6%, *n* = 124), fever or feverishness (84.3%, *n* = 118), and headache (80%, *n* = 112) (Table 1). Three specimens with co-infections of influenza A(H1N1)pdm09 and influenza A(H3N2) were omitted from the analyses. One individual had a coinfection of influenza A(H1N1)pdm09 clade 6B.1A.5a.2a.1 and SARS-CoV-2 lineage JN.1. Diarrhea (20%) and loss of sense of taste or smell (22.1%) were the least experienced symptoms.

### 3.2. Phylogenetic Analysis of Influenza Viruses

Phylogenetic analyses of the HA gene (*n* = 128) showed that the Arizona sequences were primarily influenza A(H1N1)pdm09 clades 6B.1A.5a.2a.1 (48.4%, *n* = 62) and 6B.1A.5a.2a (10.2%, *n* = 13), A(H3N2) clade 3C.2a1b.2a.2a.3a.1 (18%, *n* = 23), and B/Victoria clade V1A.3a.2 (23.4%, *n* = 30) (Figure 1B–D). We did not detect influenza B/Yamagata lineages. Based on their vaccination status in the time since 1 July 2023, 119 (85.0%) of sequenced cases report not having received an influenza or COVID-19 vaccination, while 8 (5.7%) report having received both vaccinations, 8 (5.7%) report having received only a COVID-19 vaccination, 3 (2.1%) report having received only an influenza vaccination, and 2 (1.4%) reported that they did not know whether they had received either vaccination. Of the influenza vaccinated individuals, eight were infected with influenza A(H1N1)pdm09 clade 6B.1A.5a.2a.1, one influenza A(H3N2) clade 3C.2a1b.2a.2a.3a.1, and two influenza B/Victoria clade V1A.3a.2.

### 3.3. HA and NA Mutations Identified in Circulating 2023–2024 Influenza Viruses

**Figure 2:**
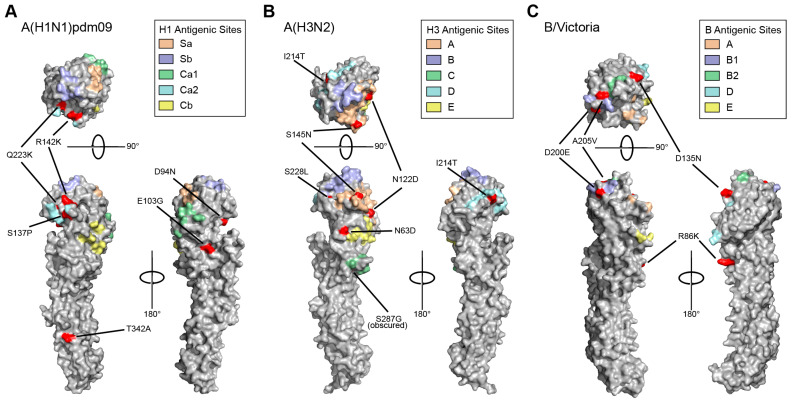
Structure of influenza HA proteins, and locations of important residues. Mutations predicted by FluSurver as interest levels 2 and 3 (red), and antigenic sites (shaded color regions) were mapped to representative crystal structures for (**A**) A(H1N1)pdm09, (**B**) A(H3N2), and (**C**) B/Victoria proteins (PDB: 7KNA, 4WE8, 4FQM).

Using the recommended numbering scheme for influenza HA subtypes [14], we compared the Arizona sequences to the cell culture and recombinant-based WHO vaccine reference viruses and identified 56 non-synonymous mutations in A(H1N1)pdm09 HA gene sequences, 33 in A(H3N2), and 18 in B/Victoria (Supplementary Tables S1–S3). Of these mutations, six A(H1N1)pdm09, six A(H3N2) and five B/Victoria mutations were predicted to be of high phenotypic consequence (FluSurver tool [18] interest levels 2 and 3). We mapped these mutations of interest to the antigenic sites of influenza HA protein crystal structures (Figure 2). The mutations were surface-exposed, and many were in or adjacent to known antigenic sites. The only level-three mutation, D94N, in H1N1 sequences was found in only five influenzas A(6B.1A.5a.2a) samples in our study, and these samples clustered separately from the rest of the 6B.1A.5a.2a sequences in the phylogenetic tree. We analyzed the influenza genome sequences from vaccinated individuals and found that there were no mutations uniquely conserved in vaccine breakthrough cases.

We also searched for mutations in the NA protein sequences and found 35 non-synonymous mutations in A(H1N1)pdm09 NA gene sequences, 18 in A(H3N2), and 19 in B/Victoria. Of these mutations, two mutations in A(H1N1) and one in Victoria were predicted to be of high phenotypic consequence (FluSurver tool interest levels 2 and 3). We did not find any high-interest mutations in A(H3N2) in our samples. One non-synonymous mutation S247N in A(H1N1)pdm09 neuraminidase sequences (4.1%) was predicted to have potential antiviral impact. No unique mutations in the NA genes for influenza-vaccinated yet influenza-infected individuals were found.

## 4. Discussion

Here, we present the genomic surveillance and analysis of influenza viruses in Arizona, USA, using next generation sequencing (NGS) and bioinformatics. We show that the influenza viruses circulating in the 2023–24 season shifted towards the A(H1N1)pdm09 subtype as compared to the predominantly A(H3N2) in the prior season. We identified the emergence of surface-exposed mutations in HA-protein antigenic sites. Our findings highlight the continued adaptive evolution of influenza viruses. Leveraging next-generation sequencing and bioinformatics, this genomic surveillance approach can be implemented for other pathogens of public health concern and pandemic/epidemic potential, for example, by monitoring circulating respiratory syncytial viruses for mutations that may impact vaccines and monoclonal antibody treatments [20]. By pivoting local sequencing capacity to monitor other public health threats, NGS infrastructure can be maintained in the absence of a pandemic emergency.

Next-generation sequencing has expanded the amount of information and details that can be used to conduct thorough and sensitive bioinformatics analysis, allowing us to identify variants across the whole genome. A limitation of this study is the sequencing selection criteria of specimens with RT-qPCR assay cycle threshold values of less than 30 may display bias towards influenza infections with high viral loads. Whether the changes identified in HA antigenic site regions are functional, immune escape adaptive mutations will need to be addressed in future studies. Further studies are also needed to determine if the high-interest-level mutation in NA can also result in functionally strong drug resistance. Annual vaccination is the most effective method in preventing and reducing the impact of influenza [21]. Vaccinated individuals tend to have reduced severity of illness, and we also did not observe common mutations unique to vaccinated individuals in our study that could explain their susceptibility to infection [22]. Nonetheless, this demonstrates the need to apply pathogen genomic sequencing to monitor vaccine breakthrough infections. Through collaborative sharing of data and information, this can lead to early detection, public health decision-making, and action.

## 5. Conclusions

Overall, this study highlights the importance of genomic sequencing surveillance for clinical and public health decisions. We found multiple high-level-of-interest mutations in HA and NA genes, of which some unique mutations could potentially cause immune escape and drug resistance in the circulating Arizona influenza strains. We demonstrate the application of next-generation sequencing and bioinformatics analyses for pathogen genomic surveillance in local communities.

## Figures and Tables

**Table 1:** Symptoms experienced.

Symptoms	Yes	No	Do Not Know/Unknown
Fever/feverishness	84.3%	11.4%	4.3%
Chills	77.1%	21.4%	1.4%
Sore throat	77.1%	21.4%	1.4%
Difficulty breathing/shortness of breath	60.0%	40.0%	0.0%
Runny nose or nasal congestion	88.6%	11.4%	0.0%
Decreased/complete loss of sense of taste or smell	22.1%	75.0%	2.9%
Headache	80.0%	20.0%	0.0%
Wheezing	37.9%	60.7%	1.4%
Nausea/vomiting	47.9%	51.4%	0.7%
Diarrhea	20.0%	79.3%	0.7%
Muscle pain or body aches	77.1%	22.1%	0.7%
Fatigue	89.3%	10.0%	0.7%

## Data Availability

Influenza genome sequences have been deposited to the GISAID repository under accession numbers EPI_ISL_18928538, EPI_ISL_18928541, EPI_ISL_18930210-18930347.

## Supplementary Materials

The following supporting information can be downloaded at: https://www.mdpi.com/article/10.3390/v16050692/s1, Table S1: A(H1N1)pdm09 HA non-synonymous mutations; Table S2: A(H3N2) HA non-synonymous mutations; Table S3: B/Victoria HA non-synonymous mutations.
