# Supplementary material for: Influenza Virus Genomic Surveillance, Arizona, USA, 2023–2024
Source: Viruses. 2024 Apr 27;16(5):692. doi: 10.3390/v16050692 (PMC11125580; doi:10.3390/v16050692)
Supplement: Supplementary file 1 [file viruses-16-00692-s001.zip › Supplementary Tables.pdf]

Supplementary Tables

Supplementary Table S1: Frequency of non-synonymous amino acid substitutions in influenza A(H1N1)pdm09 virus HA gene found in Arizona genome sequences compared to cell culture and recombinant-based WHO vaccine reference Influenza A/Wisconsin/67/2022(H1N1)pdm09 (OQ203982).

Mutation	Antigenic site	Frequency Unvaccinated (n=67)	Frequency Vaccinated (n=8)
D35E		1.5%	0.0%
D35Y		1.5%	0.0%
N38D		3.0%	0.0%
R45K		40.3%	62.5%
V47I		3.0%	0.0%
E68K		1.5%	0.0%
S69P		3.0%	0.0%
A73T		1.5%	0.0%
R74I		1.5%	0.0%
N84S		3.0%	0.0%
S85P		1.5%	0.0%
D94N	-	7.5%	0.0%
I96T		1.5%	0.0%
I96V		1.5%	0.0%
E103G	-	1.5%	0.0%
R113K		16.4%	12.5%
I116M		1.5%	0.0%
T120A		17.9%	0.0%
N130K		1.5%	0.0%
S137P	Ca2	17.9%	0.0%
R142K	Ca2	13.4%	0.0%
F144L		1.5%	0.0%
K146E		1.5%	0.0%
K169Q		4.5%	0.0%
G170E		0.0%	12.5%
A216T		14.9%	0.0%
Q223K	Ca2	13.4%	0.0%

R223Q		76.1%	62.5%
E260D		19.4%	0.0%
A261T		1.5%	0.0%
A261V		1.5%	0.0%
A277T		20.9%	0.0%
I286M		0.0%	12.5%
V295I		1.5%	0.0%
K302E		7.5%	12.5%
S309R		1.5%	0.0%
T342A	-	3.0%	0.0%
D356E		19.4%	0.0%
D356N		1.5%	0.0%
I404V		0.0%	12.5%
F415L		1.5%	0.0%
V418I		7.5%	0.0%
V427I		17.9%	12.5%
T434I		1.5%	0.0%
D436N		1.5%	0.0%
R450K		1.5%	0.0%
H451N		19.4%	0.0%
K454R		1.5%	0.0%
K480R		4.5%	0.0%
D501N		1.5%	0.0%
I510V		1.5%	0.0%
L526M		1.5%	0.0%
I533V		7.5%	0.0%

Supplementary Table S2: Frequency of non-synonymous amino acid substitutions in influenza A(H3N2) virus HA gene found in Arizona genome sequences compared to cell culture and recombinant-based WHO vaccine reference Influenza A/Darwin/6/2021(H3N2) (OQ718999).

Mutation	Antigenic site	Frequency Unvaccinated (n=22)	Frequency Vaccinated (n=1)

I3L		4.5%	0.0%
S9G		9.1%	0.0%
P21L		4.5%	0.0%
I25V		9.1%	0.0%
G62E		13.6%	0.0%
N63D	E	9.1%	0.0%
R92G		4.5%	0.0%
S95G		4.5%	0.0%
S95N		4.5%	0.0%
Y105H		4.5%	0.0%
N122D	A	90.9%	0.0%
S145N	A	13.6%	0.0%
I195F		100.0%	100.0%
K207R		18.2%	0.0%
A212S		4.5%	0.0%
I214T	B	4.5%	0.0%
N216D		4.5%	0.0%
I223V		4.5%	0.0%
S228L	B	4.5%	0.0%
I260M		4.5%	0.0%
K276E		86.4%	100.0%
S287G	-	4.5%	0.0%
N298S		4.5%	0.0%
V309I		4.5%	0.0%
V347M		9.1%	0.0%
E390K		4.5%	0.0%
V413I		4.5%	0.0%
I418V		4.5%	0.0%
K452R		4.5%	0.0%
V505I		4.5%	0.0%
I515V		4.5%	0.0%
M536V		4.5%	0.0%

Supplementary Table S3: Frequency of non-synonymous amino acid substitutions in influenza B/Victoria virus HA gene found in Arizona genome sequences compared to cell culture and recombinant-based WHO vaccine reference Influenza B/Austria/1359417/2021 (B/Victoria) (EPI_ISL_2378894).

Mutation	Antigenic site	Frequency Unvaccinated (n=28)	Frequency Vaccinated (n=2)
V30I		3.6%	0.0%
R86K	-	3.6%	0.0%
V93A		10.7%	0.0%
E134G		3.6%	0.0%
E134K		32.1%	50.0%
D135N	-	7.1%	0.0%
M162L		14.3%	0.0%
T185A		0.0%	50.0%
E186K		96.4%	50.0%
D200E	B1	96.4%	100.0%
E201G		3.6%	0.0%
E201K		17.9%	0.0%
A205V	B1	10.7%	0.0%
V255M		3.6%	0.0%
W277R		10.7%	0.0%
R501K		3.6%	0.0%
N534H		3.6%	0.0%
